# Clinical characteristics and prognosis of liver injury in patients receiving immune checkpoint inhibitor-based therapy: a real-world retrospective study

**DOI:** 10.3389/fphar.2026.1957181

**Published:** 2026-10-26

**Authors:** Liang Gao, Xiaotian Yang, Fengkun Yang, Guiling Shi, Yuan Jiang, Zhenying Zhao, Meisa Wang, Yuyang Gao, Tao Han

**Affiliations:** 1 Department of Pharmacy, Tianjin Union Medical Center, The First Affiliated Hospital of Nankai University, Tianjin, China; 2 School of Medicine, Nankai University, Tianjin, China; 3 Department of Gastroenterology and Hepatology, Tianjin Union Medical Center, The First Affiliated Hospital of Nankai University, Tianjin, China

**Keywords:** clinical characteristics, immune checkpoint inhibitors, liver injury in patients receiving ICI-based therapy, prognosis, real-world retrospective study

## Abstract

**Aims:**

This study aimed to examine the clinical characteristics, injury patterns and 6-month recovery of liver injury in patients receiving immune checkpoint inhibitor -based therapy.

**Methods:**

We conducted a retrospective study of patients receiving ICIs at Tianjin Union Medical Center (The First Affiliated Hospital of Nankai University) between January 2020 and June 2024, with follow-up censored December 2024 and a minimum 6 months. We analysed the clinical characteristics, severity, dynamic changes in liver function, and recovery factors of patients with ICI-based therapy-related liver injury (ICI-LI).

**Results:**

Of 682 patients, 83 (12.17%) developed ICI-LI (cholestatic 73.49%, mixed 20.48%, hepatocellular 6.02%). Among 83 patients, baseline alkaline phosphatase (ALP), gamma-glutamyl transferase, and total bilirubin, and peak alanine aminotransferase (ALT) and aspartate aminotransferase (AST) trend to be higher with greater liver injury severity. The rate of change in liver function parameters differed by subtype: hepatocellular injury showed rapid ALT/AST increases, cholestatic injury a marked but slower ALP increase, and mixed injury features of both. In univariate analysis, age, peak ALT, and neutrophil-to-lymphocyte ratio (NLR) > 3 were associated with poor 6-month recovery (*P* < 0.05); in multivariate analysis, only NLR >3 remained associated (OR = 10.917, 95% CI 1.552-76.817, *P* = 0.016). ROC analysis yielded AUCs of 0.702 for continuous NLR (cut-off 2.955, rounded to 3) and 0.694 for NLR >3.

**Conclusion:**

Cholestatic injury was the predominant subtype with PD-1/PD-L1 inhibitor-containing therapy. Changes in liver function parameters varied according to the clinical pattern. NLR >3 warrants further investigation as a potential exploratory biomarker for poor recovery in ICI-LI.

## 1 Introduction

Over the past decade, immune checkpoint inhibitors (ICIs) have become a cornerstone of cancer management and have substantially changed the prognosis of various advanced solid and haematological malignancies (Hountondji et al., 2023; Bagchi et al., 2021). Unlike conventional chemotherapy, which directly targets tumor cells, ICIs suppress tumors by restoring immune function. However, this mechanism can also result in immune-related adverse events (irAEs) affecting multiple organ systems. These irAEs include immune-mediated liver injury, hypothyroidism, immune-related pneumonitis, and immune-related myocardial injury (Haanen et al., 2022; Yin et al., 2023). ICI-related liver injury is a clinically significant complication, with reported incidence ranging from 0.67% to 37% (Kobayashi et al., 2023; Shah et al., 2020; Shojaie et al., 2021; Riveiro-Barciela and De Martin, 2025). ICIs were most often used in combination with other antineoplastic agents as part of a treatment regimen administered concurrently within a treatment course. Therefore, to better reflect the complexity of real-world settings, we defined immune checkpoint inhibitor-induced liver injury as liver injury in patients receiving ICI-based therapy (ICI-LI) in this study (Arie et al., 2020). Notably, the risk of ICI-LI increases further when checkpoint inhibitors are combined with other ICIs or chemotherapy agents (Shojaie et al., 2021; Kim et al., 2013). Liver injury arising during cancer treatment not only affects subsequent treatment decisions, but may also lead to hepatic failure or death in patients (Li et al., 2022). Therefore, ICI-LI presents a significant challenge in oncology. This study aimed to characterize the clinical features and prognostic determinants of ICI-LI, thereby providing insights into its prevention and clinical management.

## 2 Methods

### 2.1 Patients and data collection

We conducted a retrospective study of patients who received ICIs between January 2020 and June 2024 at the Tianjin Union Medical Center (First Affiliated Hospital of Nankai University). The last patient was enrolled on 21 June 2024, and follow-up data were censored in December 2024. Follow-up was conducted using electronic medical records or telephone interviews, and all patients were followed for at least 6 months or until death. A total of 682 patients were screened, and after rigorous screening, 83 patients were included in the final analysis, and the screening process is detailed in Figure 1.

**FIGURE 1:**
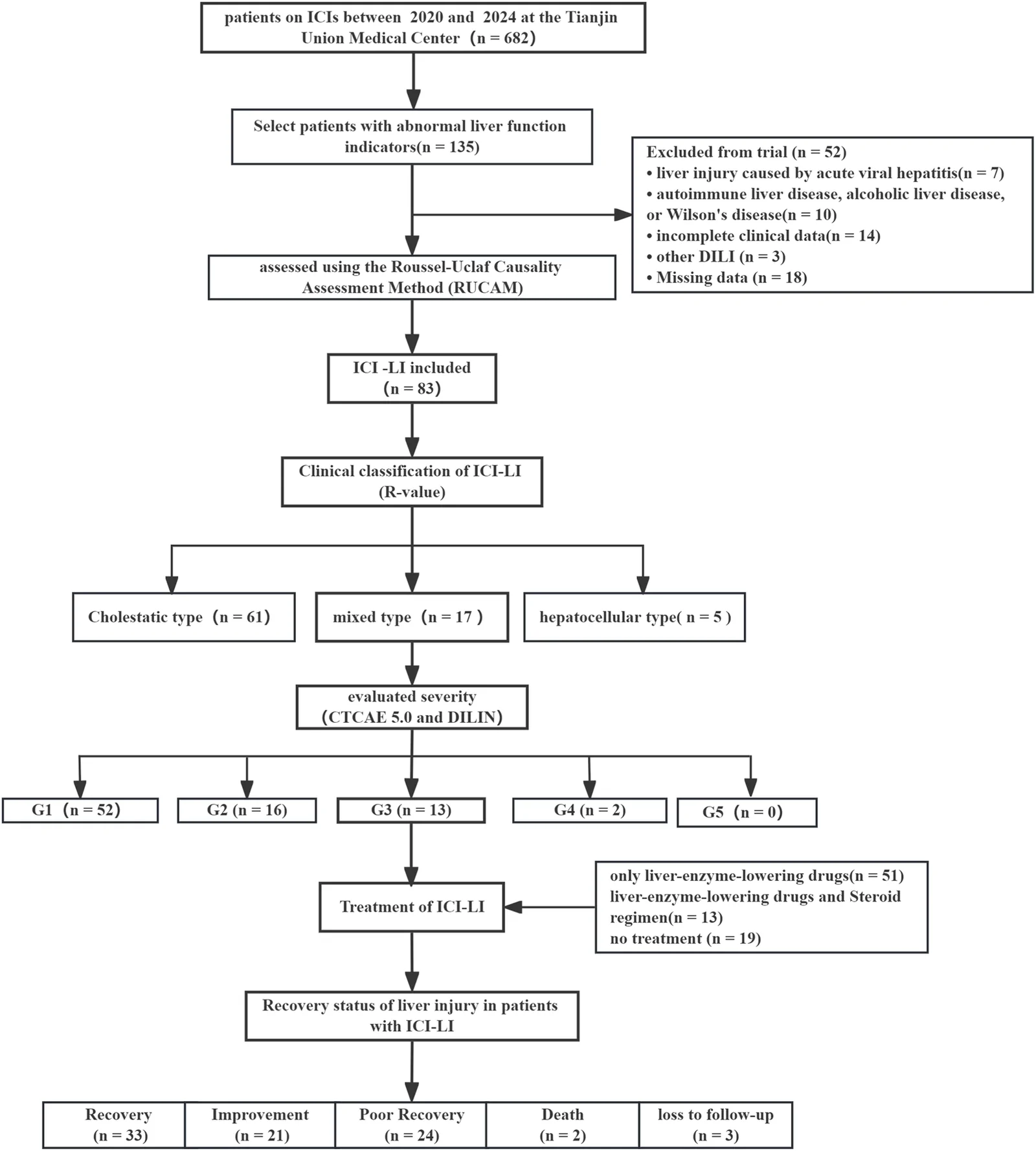
Flowchart of screening, analysis, and outcome management for the 83 patients. CTCAE, Common Terminology Criteria for Adverse Events; DILI, drug-induced liver injury; DILIN, Drug-Induced Liver Injury Network; G1, grade 1; G2, grade 2; G3, grade 3; G4, grade 4; G5, grade 5; ICI-LI, liver injury in patients receiving immune checkpoint inhibitor-based therapy; ICIs, immune checkpoint inhibitors; RUCAM, Roussel-Uclaf Causality Assessment Method.

ICI-LI was diagnosed according to the Common Terminology Criteria for Adverse Events (CTCAE), version 5.0 and the Drug-Induced Liver Injury Network (DILIN) criteria (Savino et al., 2024). The diagnostic thresholds differed depending on whether baseline liver function tests were normal or elevated, as detailed in Supplementary Table S2. Alternative aetiologies (viral, autoimmune, alcoholic, and biliary obstruction) were excluded by serological testing and abdominal imaging. According to the principles for evaluating adverse drug reactions/events (Haanen et al., 2022; Brennan et al., 2022), causality was assessed using the Roussel-Uclaf Causality Assessment Method (RUCAM) (Danan and Teschke, 2015), and only cases with a RUCAM score ≥3 were included.

### 2.2 Clinical classification of ICI-LI

According to the DILI assessment criteria formulated by the Council for International Organizations of Medical Sciences (CIOMS) in 2020 (CIOMS, 2020), the clinical classification of DILI uses the R-value (R = (ALT/ULN)/(ALP/ULN)). It is categorised as cholestatic (R ≤ 2), hepatocellular (R ≥ 5), or mixed (2 < R < 5).The R value was calculated using ALT and ALP values from a single blood draw on the day of most severe hepatic injury (the peak day), defined as the day with the highest CTCAE grade. On this day, all liver function parameters, including ALT, AST, ALP, GGT, and TBIL, were extracted simultaneously from the same blood draw.

### 2.3 Severity of ICI-LI

We evaluated the severity using the Common Terminology Criteria for Adverse Events (CTCAE) version 5.0 and Drug-Induced Liver Injury Network (DILIN) criteria (Savino et al., 2024). The details are shown in Supplementary Table S2. The final severity grade for each patient was the higher grade derived from either system.

### 2.4 ICI-LI prognosis grouping

Based on recovery status, patients were categorised into four prognostic groups: Recovery, Improvement, Poor Recovery, and Death. The definitions of each group are as follows: ①Recovery: Biochemical parameters (ALT, AST, ALP, and TBIL) returned to within the normal range, accompanied by marked or complete resolution of clinical symptoms. ②Improvement: Major biochemical parameters decreased by more than 50% from baseline and fell below 2 × ULN but remained above the normal range; clinical symptoms showed partial improvement. ③Poor Recovery: Major biochemical parameters showed no significant change; clinical symptoms showed little improvement or worsened (e.g., increasing biochemical parameters or the occurrence of severe complications). ④Death: Death due to liver failure or other DILI-related complications. For the primary prognostic analysis, patients were further dichotomised into favourable recovery (Recovery and Improvement) and poor recovery (Poor Recovery and Death).

### 2.5 Statistical methods

The Shapiro-Wilk test was used to assess the normality of the continuous variables. Normally distributed data are presented as mean ± standard deviation and were compared between groups using one-way analysis of variance. Non-normally distributed quantitative data are presented as medians (Q25, Q75) and were analysed using the Kruskal–Wallis test. Categorical data are expressed as frequencies (proportions or rates), and intergroup comparisons were performed using Fisher’s exact chi-square test.

To identify independent predictors of recovery, univariate and multivariate logistic regression analyses were performed. Variables with *P* < 0.05 in the univariate analysis were entered into the multivariate model. The prognostic value of the neutrophil-to-lymphocyte ratio (NLR) was further evaluated using receiver operating characteristic (ROC) curve analysis, and the optimal cut-off was determined using the Youden index. For clinical utility, NLR was also dichotomized at a rounded integer threshold. Given the limited sample size, these analyses are intended as exploratory and supportive. All statistical analyses were conducted using SPSS version 26.0 (IBM Corporation, Armonk, NY, United States of America).

## 3 Results

### 3.1 Patient baseline characteristics

From January 2020 to June 2024, 682 patients received ICI treatment at the hospital. After rigorous screening, 83 patients were finally included in this study cohort, and the screening process is detailed in Figure 1. The overall incidence of ICI-LI was 12.17% (83/682). Higher incidence rates were observed for individual agents: tislelizumab (13.97%, 38/272), camrelizumab (11.52%, 19/165), and sintilimab (9.90%, 19/192). The baseline characteristics of the participants are shown in Table 1.

**TABLE 1:** Baseline characteristics of patients with ICI-LI.

Variable category	Subcategory	Value, N = 83
Age (years), median (Q25, Q75)	-	61 (54.5, 68)
Sex, n (%)	Male	55 (66.27)
	Female	28 (33.73)
Lifestyle habits, %	Alcohol consumption	23 (27.71)
Medical history, %	Type 2 diabetes mellitus	6 (7.23)
	Gallbladder disease	4 (4.82)
	Splenomegaly	4 (4.82)
	Infection	5 (6.02)
	Cerebral infarction	1 (1.20)
	Hyperbilirubinemia	1 (1.20)
Underlying liver disease, %	Liver cirrhosis	5 (6.02)
	Chronic viral hepatitis	5 (6.02)
Cancer type, %	Lung cancer	40 (48.19)
	Esophageal cancer	14 (16.87)
	Colon cancer	12 (14.46)
	Liver cancer	6 (7.23)
	Gastric cancer	5 (6.02)
	Ovarian cancer	1 (1.20)
	Nasopharyngeal carcinoma	1 (1.20)
	Rectal cancer	1 (1.20)
	Bladder cancer	1 (1.20)
	Laryngeal cancer	1 (1.20)
	Vaginal malignant melanoma	1 (1.20)
Immune checkpoint inhibitors, %	Tislelizumab	38 (45.78)
	Camrelizumab	19 (22.89)
	Sintilimab	19 (22.89)
	Cadonilimab	4 (4.82)
	Envafolimab	1 (1.20)
	Toripalimab	2 (2.41)
RUCAM, %	Possible (3-5)	29 (34.94)
	Probable (6-8)	51 (61.45)
	Highly probable (≥9)	3 (3.61)
Severity grade, %	Grade 1	52 (62.65)
	Grade 2	16 (19.28)
	Grade 3	13 (15.66)
	Grade 4	2 (2.41)
	Grade 5	0 (0)
Baseline laboratory parameters median, (Q25, Q75)	ALT (U/L)	24.70 (13.14, 33.65)
	AST (U/L)	22.00 (18.15, 35.50)
	ALP (U/L)	95.00 (74.45, 130.35)
	GGT (U/L)	40.75 (28.00, 96.48)
	PLT (×10^9^/L)	190.50 (135.50, 255.00)
	Cr (μmol/L)	59.00 (50.00, 71.00)
	TBIL, (μmol/L)	12.33 (9.55, 17.05)
	ALB, (g/L)	41.30 (36.90, 43.28)
	PT, (s)	12.80 (12.00, 13.90)
	INR	1.04 (0.99, 1.10)

ALP, alkaline phosphatase; ALT, alanine aminotransferase; AST, aspartate aminotransferase; GGT, gamma-glutamyl transferase; PLT, platelet count; Cr, creatinine; TBIL, total bilirubin; ALB, albumin; PT, prothrombin time; INR, international normalised ratio; ULN, upper limit of normal; Q25, 25th percentile; Q75, 75th percentile; RUCAM, Roussel-Uclaf Causality Assessment Method.

As summarised in Table 1, the median age of the cohort was 61 years, with a predominance of males (male-to-female ratio, 1.96:1; 66.27% male). Seven patients had a prior diagnosis of liver cirrhosis or chronic viral hepatitis, and three had both. The most common malignancy was lung cancer (n = 40, 48.19%), followed by oesophageal cancer (n = 14, 16.87%), colon cancer (n = 12, 14.46%), hepatocellular carcinoma (n = 6, 7.23%), and gastric cancer (n = 5, 6.02%). The most frequently administered ICIs were tislelizumab (n = 38, 45.78%), camrelizumab (n = 19, 22.89%), and sintilimab (n = 19, 22.89%). Regarding severity, grade 1 was the most common (n = 52, 62.65%), followed by grade 2 (n = 16, 19.28%), grade 3 (n = 13, 15.66%), and grade 4 (n = 2, 2.41%). One patient with Grade 4 received tislelizumab treatment and showed a marked increase in ALT and AST levels. Another grade 4 case was associated with a regimen containing sintilimab and was characterised by elevated total bilirubin levels.

Supplementary Table S1 summarizes chemotherapy history and liver function before ICI initiation. Twenty-three patients (27.71%) had normal liver function during prior chemotherapy and developed liver injury only after ICI initiation, and 6 (7.23%) had pre-existing injury that worsened after ICI, providing temporal evidence supporting ICI contribution in these patients. In contrast, 44 patients (53.01%) received concurrent chemotherapy at enrollment, precluding definitive attribution.

### 3.2 Clinical features of ICI-LI

Among patients with ICI-LI, the injury patterns were cholestatic in 73.49% (n = 61), mixed in 20.48% (n = 17), and hepatocellular in 6.02% (n = 5). Statistically significant differences were observed among the three groups in the baseline ALP, GGT, and TBIL levels, as well as peak ALT, AST, and ALP levels after ICI administration. No significant differences were detected in the other liver function parameters, sex, age, type of ICI, number of ICI cycles, time to ICI-LI onset, or RUCAM.

Overall, 96.39% (80/83) of the patients with ICI-LI received ICIs in combination with other anticancer drugs. The most frequently combined agents were platinum-based drugs (n = 52, 62.65%), paclitaxel (n = 30, 36.15%), and bevacizumab (n = 28, 33.73%). These regimens were primarily administered for lung, colon, and oesophageal cancers, which together accounted for 79.52% of the malignancies in this cohort. Statistical analysis revealed no significant association between the type of combined anticancer drugs and the pattern of liver injury (cholestatic, mixed, or hepatocellular injury). Table 2 shows the detailed clinical characteristics.

**TABLE 2:** Clinical characteristics of patients with ICI-LI.

Variable category	Subcategory	Cholestatic type	Mixed type	Hepatocellular injury type	*P* value
Number of cases, n (%)		61 (73.49)	17 (20.48)	5 (6.02)	​
Age (years), mean ± SD		60.55 ± 10.77	59.94 ± 11.11	68.2 ± 7.27	0.265
Sex, n (%)					0.593
​	Male	39 (63.93)	13 (76.47)	3 (60.00)	​
​	Female	22 (36.07)	4 (23.53)	2 (40.00)	​
ICIs, n (%)					0.258
​	Tislelizumab	29 (47.54)	6 (35.29)	3 (60.00)	​
​	Camrelizumab	13 (21.31)	6 (35.29)	0 (0.00)	​
​	Sintilimab	15 (24.59)	3 (17.65)	1 (20.00)	​
​	Cadonilimab	3 (4.92)	1 (5.88)	0 (0.00)	​
​	Envafolimab	0 (0.00)	1 (5.88)	0 (0.00)	​
​	Toripalimab	1 (1.64)	0 (0.00)	1 (20.00)	​
Number of ICI infusions, median (Q25, Q75)		2 (1, 5)	2 (1, 4.5)	2 (1, 2.5)	0.650
Liver function tests, median (Q25, Q75)					
*Baseline*	ALT (U/L)	23.00 (12.40, 30.45)	27.00 (16.50, 37.65)	70.00 (20.05, 95.60)	0.102
	AST (U/L)	23.40 (17.70, 38.50)	22.30 (17.65, 34.50)	20.00 (16.75, 23.30)	0.561
	ALP (U/L)	110.40 (83.30, 136.75)	77.00 (66.85, 122.00)	24.00 (12.50, 87.30)	0.003
	GGT (U/L)	62.10 (28.03, 129.85)	34.10 (27.90, 44.67)	22.30 (19.20, 42.00)	0.048
	TBIL (μmol/L)	12.50 (9.80, 17.20)	11.00 (9.10, 15.70)	37.00 (14.90, 42.70)	0.021
*Peak*	ALT (U/L)	35.00 (16.55, 62.85)	116.00 (63.90, 211.00)	145.00 (106.05, 1536.75)	<0.001
	AST (U/L)	40.60 (26.50, 73.95)	76.90 (38.05, 129.75)	123.60 (48.00, 1322.45)	0.023
	ALP (U/L)	166.80 (129.15, 244.58)	97.00 (71.20, 156.40)	59.50 (32.50, 133.15)	<0.001
	GGT (U/L)	172.05 (64.83, 252.20)	110.00 (38.20, 178.50)	120.70 (45.90, 228.30)	0.230
	TBIL (μmol/L)	13.20 (8.93, 21.58)	11.30 (8.20, 16.20)	16.40 (10.40, 37.45)	0.369
Concomitant medications, n (%)					
Paclitaxel-containing regimen		23 (27.71)	6 (7.23)	1 (1.20)	0.853
Bevacizumab-containing regimen		22 (26.51)	5 (6.02)	1 (1.20)	0.776
Platinum-containing regimen		38 (45.78)	11 (13.25)	3 (3.61)	1.000

ALP, alkaline phosphatase; ALT, alanine aminotransferase; AST, aspartate aminotransferase; GGT, gamma-glutamyl transferase; TBIL, total bilirubin; ICI, immune checkpoint inhibitor; Q25, 25th percentile; Q75, 75th percentile; SD, standard deviation.

### 3.3 Grading of ICI-LI and analysis of clinical characteristics

According to the severity grading based on CTCAE v5.0 and DILIN criteria, the distribution of ICI-LI cases in this cohort was as follows. Cholestatic type: grades 1 (n = 40), 2 (n = 12), 3 (n = 8), and 4 (n = 1). Mixed type: Grade 1 (n = 10), 2 (n = 3), and 3 (n = 4). Hepatocellular type: grade 1 (n = 2), 2 (n = 1), 3 (n = 1), and 4 (n = 1). Because of the limited number of grade 4 cases (n = 2) and the absence of grade 5 events, grades 3-5 were consolidated into a single category termed “severe and above liver injury (grades 3-5)” to meet the statistical requirements. Therefore, comparative analyses were performed across three severity categories: grades 1, 2, and 3-5. The Fisher exact test revealed no significant differences in the proportions of the three liver injury types (cholestatic, mixed, and hepatocellular) across the severity categories (*P* > 0.05). Similarly, no significant differences were observed in the baseline liver function parameters (ALT, AST, ALP, and GGT) among the three severity groups (*P* > 0.05). Notably, the median values of baseline liver enzyme parameters tended to increase with increasing severity of liver injury (Table 3).

**TABLE 3:** Clinical characteristics of ICI-LI by severity.

Variable	Grade 1	Grade 2	Grades 3-5	*P* value
Number of cases, n (%)	52 (62.65%)	16 (19.28%)	15 (18.07%)	​
Age (years), mean ± SD	60.92 ± 10.91	61.38 ± 10.97	59.53 ± 9.23	0.876
Sex, n (%)	​	​	​	0.320
Male	36 (69.23)	8 (50.00)	11 (73.33)	​
Female	16 (30.77)	8 (50.00)	4 (26.67)	​
Liver injury type, n (%)	​	​	​	0.531
Cholestatic type	40 (76.92)	12 (75.00)	9 (60.00)	​
Mixed type	10 (19.23)	3 (18.75)	4 (26.67)	​
Hepatocellular injury type	2 (3.85)	1 (6.25)	2 (13.33)	​
History of hepatitis B, n (%)	2 (3.85)	1 (6.25)	2 (13.33)	0.185
Immune checkpoint inhibitors, n (%)	​	​	​	0.320
Tislelizumab	28 (53.85)	4 (25.00)	6 (40.00)	​
Camrelizumab	12 (23.08)	4 (25.00)	3 (20.00)	​
Sintilimab	9 (17.31)	5 (31.25)	5 (33.33)	​
Cadonilimab	1 (1.92)	2 (12.50)	1 (6.67)	​
Envafolimab	1 (1.92)	0 (0.00)	0 (0.00)	​
Toripalimab	1 (1.92)	1 (6.25)	0 (0.00)	​
Number of ICI infusion cycles median (Q25, Q75)	2.00 (1.00, 4.50)	2.00 (1.00, 7.00)	1.00 (1.00, 3.25)	0.662
Time to onset (days), median (Q25, Q75)	55.00 (30.00, 183.50)	99.50 (28.75, 302.00)	26.50 (14.25, 86.00)	0.212
Baseline laboratory tests, median (Q25, Q75)				
ALT (U/L)	23.00 (13.15, 30.50)	26.50 (13.00, 33.64)	30.60 (16.15, 57.00)	0.197
AST (U/L)	21.50 (16.15, 27.5)	29.05 (18.08, 46.95)	32.80 (18.90, 77.75)	0.115
ALP (U/L)	93.20 (72.10, 123.50)	106.53 (81.00, 150.13)	124.10 (76.00, 143.25)	0.271
GGT (U/L)	36.60 (24.45, 82.71)	54.18 (31.50, 130.25)	54.70 (24.33, 292.50)	0.444
TBIL (μmol/L)	12.40 (9.35, 17.20)	12.43 (10.25, 16.10)	13.75 (9.65, 18.93)	0.707
ALB (g/L)	41.40 (36.90, 43.55)	41.75 (37.57, 43.40)	40.95 (37.05, 43.55)	0.982
PT (s)	12.80 (12.05, 14.05)	12.95 (11.25, 13.70)	12.30 (11.90, 13.80)	0.400
INR	1.05 (1.01, 1.10)	1.00 (0.95, 1.06)	1.04 (0.99, 1.08)	0.136
Antinuclear antibody, n (%)	3 (5.77)	2 (12.50)	0 (0.00)	0.528
Adverse reactions in other systems, n (%)	17 (32.69)	5 (31.25)	5 (33.33)	1.000
Concomitant medications, n (%)				
Paclitaxel-containing regimen	20 (38.46)	4 (25.00)	6 (40.00)	0.616
Bevacizumab-containing regimen	17 (32.69)	5 (31.25)	6 (40.00)	0.893
Platinum-containing regimen	35 (67.31)	8 (50.00)	9 (60.00)	0.421

ALB, albumin; ALP, alkaline phosphatase; ALT, alanine aminotransferase; AST, aspartate aminotransferase; GGT, gamma-glutamyl transferase; ICI, immune checkpoint inhibitor; INR, international normalized ratio; PT, prothrombin time; Q25, 25th percentile; Q75, 75th percentile; SD, standard deviation; TBIL, total bilirubin.

### 3.4 Analysis of the rate of change in clinical parameters during the progression of ICI-LI

The greatest increases in ALT and AST levels occurred in the hepatocellular type, and were significantly higher than those in the mixed and cholestatic types (*P* < 0.01). Conversely, the cholestatic type showed significantly greater elevations in ALP than did the other two types (*P* = 0.020). No significant differences were observed in the changes in TBIL or international normalized ratio (INR) among the three types (*P* > 0.05).

The rates of change (Δ/day) for each parameter were calculated based on the time at which the patients’ liver function parameters reached their peak values. The hepatocellular type showed the most rapid increase in ALT, which was 3.5 times that of the mixed type and 69.3 times that of the cholestatic type (*P* < 0.001). The rate of AST increase in the hepatocellular type reached 2.22 U/L per day, which was significantly higher than that in the other two types (*P* = 0.006). No significant intergroup differences were detected in the rates of change in TBIL or INR (*P* > 0.05).

These findings indicate that hepatocellular injury is characterized by a rapid and marked increase in ALT and AST levels, whereas cholestatic injury is characterized by a more gradual increase in ALP levels. The mixed type displayed both hepatocellular and cholestatic features: the magnitude of ALT increase was comparable to that in hepatocellular injury; nonetheless, the progression rate was significantly slower. Thus, the mixed and hepatocellular types could be distinguished by the rate of ALT increase (Table 4).

**TABLE 4:** Trends in liver function parameters among patients with different types of ICI-LI.

Variable	Cholestatic type	Mixed type	Hepatocellular injury type	*P* value
Number of cases, n (%)	61 (73.49)	17 (20.48)	5 (6.02)	​
Change amount				
ΔALT (U/L)	9.60 (−3.78, 41.95)	103.00 (48.00, 148.25)	113.20 (43.50, 1464.55)	<0.001
ΔALP (U/L)	53.35 (4.10, 115.90)	3.05 (−7.85, 28.00)	20.00 (−0.35, 73.95)	0.020
ΔAST (U/L)	15.40 (−0.20, 43.50)	54.45 (19.15, 116.68)	109.90 (27.00, 1300.25)	0.010
ΔGGT (U/L)	34.90 (1.17, 155.36)	57.00 (−1.50, 83.74)	89.00 (18.20, 199.50)	0.672
ΔTBIL (μmol/L)	1.00 (−5.85, 5.08)	−1.45 (−6.36, 1.45)	−20.60 (−24.95, 15.20)	0.095
ΔINR	0.00 (−0.07, 0.05)	0.02 (−0.018, 0.09)	0.05 (−0.06, 0.15)	0.432
Change rate				
ΔALT/T (U/L per day)	0.0561 (−0.0225, 0.5403)	1.1168 (0.4808, 5.3333)	3.8889 (1.1409, 42.0933)	<0.001
ΔALP/T (U/L per day)	0.3673 (0.0562, 1.3801)	0.0329 (−0.0494, 0.9596)	0.9528 (0.6742, 2.5142)	0.172
ΔAST/T (U/L per day)	0.0981 (−0.0029, 0.8003)	0.4140 (0.1673, 3.1791)	2.2222 (1.0659, 36.5043)	0.006
ΔGGT/T (U/L per day)	0.2765 (0.0192, 1.2322)	0.3816 (−0.0600, 2.5812)	2.8528 (0.5394, 7.5808)	0.350
ΔTBIL/T (μmol/L per day)	−0.0079 (−0.0516, 0.0650)	−0.0239 (−0.0567, 0.0091)	−0.7103 (−1.1217, 0.4671)	0.117
ΔINR/T	0.0000 (−0.0006, 0.0007)	0.0002 (−0.0002, 0.0011)	0.0020 (−0.0017, 0.0046)	0.367

ALP, alkaline phosphatase; ALT, alanine aminotransferase; AST, aspartate aminotransferase; GGT, gamma-glutamyl transferase; INR, international normalized ratio; T, time point at which the parameter reaches its peak value; TBIL, total bilirubin.

### 3.5 Pharmacological treatment of ICI-LI

Ten drugs were used to treat ICI-LI in this cohort, including glucocorticoids, bicyclol, and magnesium isoglycyrrhizinate. Spontaneous remission occurred in 19 patients (22.89%) who did not receive pharmacological interventions. 26 patients (31.33%) received monotherapy, most commonly with bicyclol, diammonium glycyrrhizinate, methylprednisolone, ursodeoxycholic acid, and magnesium isoglycyrrhizinate. 19 patients (22.89%) received combination therapy with three or more drugs, mainly for mixed-type and hepatocellular ICI-LI cases. All patients with grade 3 or 4 ICI-LI received combination therapy with at least two agents. No patients received immunosuppressive treatment other than glucocorticoids. Table 5 presents the therapeutic drugs used for ICI-LI.

**TABLE 5:** Therapeutic drugs for liver injury in patients with ICI-LI.

Therapeutic drugs	Number of cases, n (%)
Methylprednisolone/dexamethasone	13 (15.66)
Reduced glutathione	32 (38.55)
Polyene phosphatidylcholine	5 (6.02)
Ursodeoxycholic acid	8 (9.64)
Ademetionine	4 (4.82)
Compound glycyrrhizin, diammonium glycyrrhizinate, magnesium isoglycyrrhizinate	37 (44.58)
Bicyclol	31 (37.35)
Silybin	3 (3.61)

### 3.6 Recovery status of liver injury in patients with ICI-LI

A comprehensive assessment of hepatic recovery was performed in 80 of the 83 patients; three patients transferred to other hospitals were lost to follow-up and were excluded from the recovery-outcome analysis (Figure 1). By the end of the follow-up period, 54 patients had recovered or shown improvement. The remaining 26 patients showed suboptimal recovery: 17 with persistent abnormal liver function, seven with worsening biochemical markers, two with deaths.

Based on recovery outcomes, patients were categorized into two groups: favorable recovery (recovery and improvement) and poor recovery (poor recovery and death). Univariate logistic regression analysis showed that age, peak ALT level, and an NLR >3 were associated with poor recovery within 6 months in patients with ICI-LI (*P* < 0.05). ICI type was not significantly associated with recovery. Multivariately, NLR >3 remained associated with poor recovery (OR = 10.917, 95%CI: 1.552-76.817, *P* = 0.016), while age and peak ALT were attenuated (*P* > 0.05). Additionally, the number of hepatoprotective agents used did not differ significantly between the outcome groups (Table 6).

**TABLE 6:** Univariate and multivariate logistic regression analyses of liver function recovery in patients with ICI-LI.

Analyzed factor	Univariate analysis			Multivariate analysis		
​	OR	95% CI	*P* value	OR	95% CI	*P* value
Sex	0.579	0.208, 1.613	0.296	​	​	​
Age	0.951	0.907, 0.998	0.039	0.929	0.861,1.003	0.061
Drinking history	0.518	0.167, 1.607	0.255	​	​	​
Peak values of liver injury laboratory parameters						
Alanine aminotransferase (ALT, U/L)	0.979	0.963, 0.994	0.008	0.98	0.960,1.001	0.056
Alkaline phosphatase (ALP, U/L)	1.000	0.995, 1.004	0.879	​	​	​
Aspartate aminotransferase (AST, U/L)	0.999	0.996, 1.002	0.515	​	​	​
γ-glutamyl transferase (GGT, U/L)	1.000	0.999, 1.002	0.650	​	​	​
Total bilirubin (TBIL, μmol/L)	0.991	0.970, 1.013	0.427	​	​	​
Albumin (ALB, g/L)	0.971	0.899, 1.049	0.460	​	​	​
Prothrombin time (PT, s)	1.259	0.878, 1.805	0.210	​	​	​
Platelet count (PLT, ×10^9^/L)	0.999	0.994, 1.004	0.699	​	​	​
Serum creatinine (Cr, μmol/L)	1.001	0.973, 1.030	0.935	​	​	​
International normalized ratio (INR)	17.017	0.141, 2047.028	0.246	​	​	​
Neutrophil-to-lymphocyte ratio (NLR) > 3	8.000	1.575, 40.632	0.012	10.917	1.552,76.817	0.016
Platelet-to-lymphocyte ratio (PLR) > 180	1.417	0.444, 4.521	0.556	​	​	​
Type of ICI	​	​	0.227	​	​	​
Tislelizumab	1.000	Reference	​	​	​	​
Camrelizumab	0.262	0.065, 1.064	0.061	​	​	​
Sintilimab	0.400	0.110, 1.459	0.165	​	​	​
Cadonilimab	4.200	0.397, 44.401	0.233	​	​	​
Number of ICI infusion cycles	1.063	0.898, 1.258	0.476	​	​	​
Onset time (days)	1.000	0.998, 1.003	0.833	​	​	​
Severity grade	​	​	0.436	​	​	​
Grade 1 (mild)	1.000	Reference	​	​	​	​
Grade 2 (moderate)	0.375	0.094, 1.499	0.165	​	​	​
Grade 3 (severe)	0.450	0.110, 1.841	0.267	​	​	​
Grade 4 (life-threatening)	−^1^	-	-	​	​	​
Number of therapeutic drugs	​	​	0.328	​	​	​
No treatment	1.000	Reference	​	​	​	​
Monotherapy	1.122	0.322, 3.908	0.856	​	​	​
Two-drug combination therapy	0.725	0.187, 2.809	0.642	​	​	​
Multidrug combination therapy	0.295	0.062, 1.395	0.124	​	​	​
Occurrence of other adverse reactions	0.626	0.224, 1.750	0.372	​	​	​

−^1^, For the grade 4 severity group, the sample size was too small (n = 2) and the prognosis was favorable; therefore, an interpretable OR, value could not be calculated.

Reference categories were as follows: Sex (Male), ICI, type (Tislelizumab), severity grade (Grade 1), and number of therapeutic drugs (No treatment). OR > 1 indicates increased odds of poor recovery.

ALB, albumin; ALP, alkaline phosphatase; ALT, alanine aminotransferase; AST, aspartate aminotransferase; CI, confidence interval; Cr, creatinine; GGT, gamma-glutamyl transferase; ICI, immune checkpoint inhibitor; INR, international normalized ratio; NLR, neutrophil-to-lymphocyte ratio; OR, odds ratio; PLR, platelet-to-lymphocyte ratio; PLT, platelet count; PT, prothrombin time; TBIL, total bilirubin.

To further evaluate the predictive performance of NLR for 6-month poor recovery, ROC curve analysis was performed. Continuous NLR yielded an area under the curve (AUC) of 0.702 (Figure 2), and the Youden index identified an optimal cut-off value of 2.955. For clinical applicability, this cut-off was rounded to 3 for subsequent analyses. When NLR was dichotomized at the clinically practical threshold of NLR >3, the AUC was 0.694. These results support the use of NLR >3 as a potential exploratory biomarker.

**FIGURE 2:**
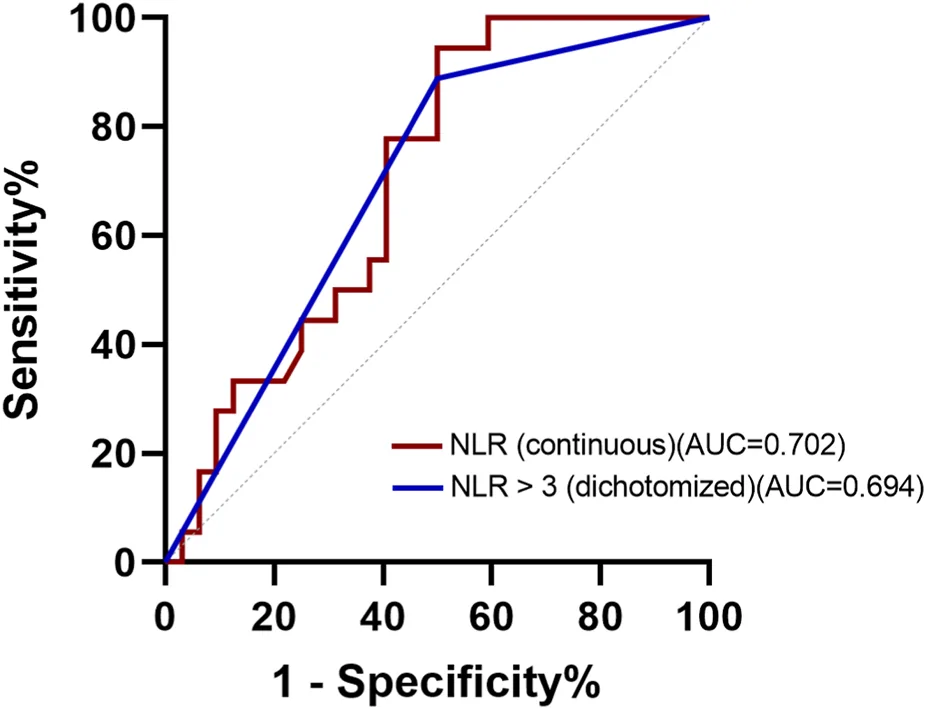
Receiver operating characteristic (ROC) curve of NLR for predicting 6-month poor recovery in patients with ICI-LI. The area under the curve (AUC) was 0.702 (95% CI: 0.560-0.844, *P* = 0.019). The optimal cut-off value determined by the Youden index was 2.955, which was rounded to 3 for clinical applicability. NLR, neutrophil-to-lymphocyte ratio; CI, confidence interval; AUC, area under the curve.

## 4 Discussion

In this retrospective observational study, 83 patients who met the diagnostic criteria for ICI-LI were identified from a cohort of 682 patients treated with ICI. The incidence of ICI-LI was 12.17%, consistent with previously reported ranges (0.67%–37%) (Imoto et al., 2019; Remash et al., 2021; Miah et al., 2023).

According to the clinical classification criteria, ICI-LI cases were categorised into three subtypes: cholestatic, hepatocellular, and mixed. The cholestatic subtype was the most prevalent (73.49%), followed by mixed (20.48%) and hepatocellular (6.02%) subtypes. The predominance of the cholestatic pattern in our cohort may be explained by the homogeneity of treatment exposure: 95.18% (79/83) of patients received PD-1/PD-L1 inhibitors, and only four patients (4.82%) received the PD-1/CTLA-4 bispecific antibody cadonilimab. Evidence links cholestatic injury specifically to PD-1/PD-L1 inhibitors, whereas hepatocellular injury is more frequently associated with anti-CTLA-4 agents. A study of 56 patients with severe ICI-LI (Imoto et al., 2019) reported cholestatic or mixed injury in 60.7% of all-grade cases (hepatocellular 19.6%), with cholestatic/mixed injury more frequent under anti-PD-1/PD-L1 monotherapy than under anti-CTLA-4-containing regimens. Similarly, a multicentre retrospective cohort of 117 ICI-LI patients (Hountondji et al., 2023) reported a cholestatic pattern in 36.8% of cases, the majority of whom had received PD-1 inhibitors (88.9%), and further revealed that hepatocellular injury was significantly associated with anti-CTLA-4 inhibitor use, whereas the cholestatic pattern was linked to PD-1/PD-L1 inhibitor therapy (Hountondji et al., 2023; Finn et al., 2020; Pi et al., 2021; Stein et al., 2021).

Statistically significant differences in baseline ALP, GGT, and TBIL levels were observed among the three. In this study, 96.39% of the patients received ICIs in combination with other antineoplastic agents. Given the predominance of colon and lung cancers in this cohort, the most frequent concomitant agents were platinum-based drugs (n = 52, 62.65%), paclitaxel (n = 30, 36.15%), and bevacizumab (n = 28, 33.73%). Statistical analysis revealed no significant association between combination regimens and clinical subtypes of ICI-LI.

According to previous reports, elevated AST and/or ALT levels are the primary features of hepatocellular ICI-LI, with an incidence ranging from 15% to 23% across all grades and 6%–13% for grade 3–4 events. In contrast, cholestatic injury is characterized by increased ALP and GGT levels (De Martin et al., 2018). However, previous studies have not systematically analyzed the dynamics of these changes. In the present study, the rate of change in liver function parameters differed among subtypes: hepatocellular ICI-LI was characterised by a rapid and marked rise in ALT and AST; cholestatic ICI-LI by a marked elevation in ALP at a relatively slow rate; and mixed ICI-LI by features of both, with the magnitude of ALT elevation comparable to that of hepatocellular injury but a significantly slower progression rate. As only five hepatocellular-type cases were included, the estimated rates of change for this subgroup are unstable and require confirmation in larger cohorts.

Regarding pharmacotherapy in this study, 22.89% of patients received no treatment and yet achieved spontaneous recovery. Additionally, 31.33% of the patients received single-agent therapy, most commonly bicyclol, diammonium glycyrrhizinate, methylprednisolone, or ursodeoxycholic acid. Notably, 22.89% of patients received combination therapy with three or more drugs, mainly for mixed and hepatocellular ICI-LI. The number of hepatoprotective drugs administered was not significantly associated with recovery outcomes (*P* > 0.05). A corticosteroid-based regimen was administered to 15.66% of the patients. Ursodeoxycholic acid and adenosylmethionine were used in 9.64% and 4.82% of patients, respectively. Despite the high prevalence of cholestatic ICI-LI (73.49%), the utilisation of targeted medications for this subtype was relatively low. Some patients with mild ICI-LI may follow a self-limiting course and do not require aggressive pharmacological interventions. This observation highlights the importance of avoiding additional liver injury that may result from the overtreatment. For the individualised management of ICI-LI, clinical practice guidelines (Haanen et al., 2022; Schneider et al., 2021) should be followed, with comprehensive consideration of liver injury grade, tumour prognosis, and the urgency of continuing immunotherapy.

Previous studies (Lee et al., 2021; Zhao et al., 2022) have proposed the NLR as a biomarker of systemic immune inflammation and a predictor of irAE. In exploratory analyses, NLR >3 was associated with poor recovery in patients with ICI-LI (OR = 10.917, 95% CI: 1.552-76.817, *P* = 0.016). ROC analysis supported this finding, with an AUC of 0.702 for continuous NLR and an optimal cut-off of 2.955, closely matching the clinically practical threshold of NLR >3 (AUC = 0.694) (Figure 2), indicating moderate discriminative ability. Nevertheless, the wide confidence interval of the OR reflects limited statistical power due to the small sample size; therefore, while ROC analysis supports the biological plausibility and clinical applicability of the NLR >3 threshold, this finding should still be considered preliminary and requires validation in larger, multicentre cohorts.

Several limitations should be acknowledged. This single-centre retrospective study predominantly involved PD-1/PD-L1 inhibitors, with only four patients receiving PD-1/CTLA-4 bispecific antibody cadonilimab, limiting generalisability. Moreover, 96.39% of patients received concomitant hepatotoxic chemotherapy or targeted therapy; despite RUCAM assessment and exclusion of alternative causes, the observed hepatotoxicity may reflect combined drug toxicity rather than ICIs alone. Our findings therefore pertain to ICI-based therapeutic-related liver injury, and the attributable risk of ICIs alone remains uncertain. Finally, given the small hepatocellular subgroup, limited sample size, wide confidence intervals, and lack of calibration or external validation, the prognostic findings should be considered exploratory and require confirmation in larger, multicentre, prospective studies.

In conclusion, this study systematically analysed the clinical characteristics, treatment strategies, and outcomes of ICI-LI and explored potential predictive biomarkers. Our findings indicate that cholestatic injury is the predominant subtype after PD-1/PD-L1 inhibitor-containing therapy, that the rate of change in liver function parameters has distinctive features across subtypes, and that NLR >3 warrants further investigation as a potential exploratory biomarker for poor recovery. These results provide insights that may inform the monitoring and management of liver injury in patients receiving ICI-based therapy.

## Data Availability

The data supporting the conclusions of this article are available from the corresponding author upon reasonable request, subject to institutional and ethical restrictions.
