# Supplementary material for: Clinical characteristics and prognosis of liver injury in patients receiving immune checkpoint inhibitor-based therapy: a real-world retrospective study
Source: Front Pharmacol. 2026 Oct 26;17:1957181. doi: 10.3389/fphar.2026.1957181 (PMC13745257; doi:10.3389/fphar.2026.1957181)
Supplement: Supplementary file 1 [file Supplementaryfile1.docx]

Supplementary Table 1. Chemotherapy history and liver function prior to ICI initiation

| Group | Category | Number of cases, n (%) |
| --- | --- | --- |
| 1 | Prior chemotherapy without liver injury | 23（27.71） |
| 2 | Prior chemotherapy with liver injury that did not recover before the baseline of this study and worsened after ICI therapy | 6（7.23） |
| 3 | Prior chemotherapy with liver injury that returned to normal before the baseline of this study | 3（3.61） |
| 4 | Concurrent chemotherapy with ICI (initiated simultaneously) | 44（53.01） |
| 5 | Unknown / Outside hospital data (normal liver function at baseline) | 7（8.43） |

Supplementary Table 2. Grading of hepatotoxicity by the Common Terminology Criteria of Adverse Events (CTCAE) v5.0 and Drug-Induced Liver Injury Network (DILIN)

| Grade | CTCAE | DILIN |
| --- | --- | --- |
| Grade 1 | • ALT/AST | • ALT and/or ALP elevation above ULN |
|  | >1–3 × ULN | but |
|  | >1.5–3 × baseline if baseline was abnormal | • Total bilirubin <2.5 mg/dL and INR <1.5 |
|  | • ALP/γGT |  |
|  | >1–2.5 × ULN |  |
|  | >2–2.5 × baseline if baseline was abnormal |  |
|  | • Total bilirubin |  |
|  | >1–1.5 × ULN |  |
|  | >1–1.5 × baseline if baseline was abnormal |  |
| Grade 2 | • ALT/AST | • ALT and/or ALP elevation above ULN |
|  | >3–5 × ULN | but |
|  | >3–5 × baseline if baseline was abnormal | • Total bilirubin ≥2.5 mg/dL or INR ≥1.5 |
|  | • ALP/γGT |  |
|  | >2.5–5 × ULN |  |
|  | >2.5–5 × baseline if baseline was abnormal |  |
|  | • Total bilirubin |  |
|  | >1.5–3 × ULN |  |
|  | 1.5–3 × baseline if baseline was abnormal |  |
| Grade 3 | • ALT/AST | • ↑ALT, ALP, total bilirubin, and/or INR |
|  | >5–20 × ULN | • and hospitalization due to DILI |
|  | >5–20 × baseline if baseline was abnormal | • or prolonging existing hospitalization due toDILI |
|  | • ALP/γGT |  |
|  | >5–20 × ULN |  |
|  | >5–20 × baseline if baseline was abnormal |  |
|  | • Total bilirubin |  |
|  | >3–10 × ULN |  |
|  | >3–10 × baseline if baseline was abnormal |  |
| Grade 4 | •ALT/AST | •↑ ALT and/or ALP + Total Bilirubin ≥ 2.5 mg/dL |
|  | >20 × ULN | • and at least one of the following: |
|  | >20 × baseline if baseline was abnormal | # Hepatic failure (INR ≥1.5, ascites,  orhepatic encephalopathy); |
|  | • ALP/γGT | # other organ failure believed to be due to  DILI event |
|  | >20 × ULN |  |
|  | >20 × baseline if baseline was abnormal |  |
|  | • Total bilirubin |  |
|  | >10 × ULN |  |
|  | >10 × baseline if baseline was abnormal |  |
| Grade 5 | • Death due to hepatotoxicity | • Death or liver transplant due to DILI event |

Abbreviations:ALP, alkaline phosphatase; ALT, alanine aminotransferase; AST, aspartate aminotransferase ; GGT, gamma-glutamyl transferase ; INR, international normalised ratio; ULN, upper limit of normal; DILI, druginduced liver injury ; ↑, increase.
